# Supplementary material for: Safety and Efficacy of Nusinersen and Risdiplam for Spinal Muscular Atrophy: A Systematic Review and Meta-Analysis of Randomized Controlled Trials
Source: Brain Sci. 2023 Oct 7;13(10):1419. doi: 10.3390/brainsci13101419 (PMC10605531; doi:10.3390/brainsci13101419)

Supplementary Material

Safety and Efficacy of Nusinersen and Risdiplam for Spinal Muscular Atrophy: A Systematic Review and Meta-Analysis of Randomized Controlled Trials

Supplementary Table S1. Detailed search strategy in four databases.

Database	Search strategy
Pubmed	1"Spinal Muscular Atrophies of Childhood"[MeSH Terms] OR "spinal muscular atrophy type iv"[Supplementary Concept] OR ("spinal muscular atrophy type ii"[Title/Abstract] OR "spinal muscular atrophy type ii"[Title/Abstract] OR "werdnig hoffmann disease"[Title/Abstract] OR "spinal muscular atrophy infantile"[Title/Abstract] OR "spinal muscular atrophy type i"[Title/Abstract] OR "SMA"[Title/Abstract] OR "muscular atrophy spinal"[Title/Abstract] OR "spinal muscular atrophy"[Title/Abstract] OR "juvenile spinal muscular atrophy"[Title/Abstract] OR "muscular atrophy juvenile"[Title/Abstract] OR "spinal muscular atrophy type iii"[Title/Abstract] OR "kugelberg welander disease"[Title/Abstract] OR "spinal muscular atrophy type 3"[Title/Abstract] OR (("muscular atrophy, spinal"[MeSH Terms] OR ("Muscular"[All Fields] AND "Atrophy"[All Fields] AND "Spinal"[All Fields]) OR "spinal muscular atrophy"[All Fields] OR ("Muscular"[All Fields] AND "Atrophy"[All Fields] AND "Spinal"[All Fields]) OR "muscular atrophy spinal"[All Fields]) AND "type iii"[Title/Abstract]) OR (("muscular atrophy, spinal"[MeSH Terms] OR ("Muscular"[All Fields] AND "Atrophy"[All Fields] AND "Spinal"[All Fields]) OR "spinal muscular

	atrophy"[All Fields] OR ("Spinal"[All Fields] AND "Muscular"[All Fields] AND "Atrophy"[All Fields])) AND "adult form"[Title/Abstract]) OR "spinal muscular atrophy type iv"[Title/Abstract])<br><br>2"nusinersen"[Title/Abstract] OR "ASO-10-27"[Title/Abstract] OR ("ISIS-SMN"[All Fields] AND "Rx"[Title/Abstract]) OR "ISIS-SMNRx"[Title/Abstract] OR "isis 396443"[Title/Abstract] OR "SPINRAZA"[Title/Abstract] OR "risdiplam"[Title/Abstract] OR "nusinersen"[Supplementary Concept]<br><br>3"randomized"[Title/Abstract] OR "placebo"[Title/Abstract] OR "randomized controlled trial"[Publication Type]<br><br>4 #1 AND #2 AND #3
Embase*	1 'spinal muscular atrophies of childhood'/exp<br><br>2 'werdnig hoffmann disease':ti,ab OR 'spinal muscular atrophy type i':ti,ab OR 'sma' OR 'muscular atrophy, spinal':ti,ab OR 'type i spinal muscular atrophy':ti,ab OR 'spinal muscular atrophy':ti,ab OR 'juvenile spinal muscular atrophy' OR 'spinal muscular atrophy type iii':ti,ab OR 'muscular atrophy, juvenile' OR 'kugelberg welander disease':ti,ab OR 'spinal muscular atrophy, type 3':ti,ab OR 'muscular atrophy, spinal, type iii' OR 'spinal muscular atrophy, type iv':ti,ab OR 'spinal muscular atrophy, adult form':ti,ab<br><br>3 'nusinersen'/exp<br><br>4 'nusinersen':ti,ab OR 'aso-10-27':ti,ab OR 'isis-smnrx':ti,ab OR 'isis 396443':ti,ab OR 'spinraza':ti,ab<br><br>5 'risdiplam'/exp

	6 #1 OR #2<br><br>7#3 OR #4 OR #5<br><br>8#6 AND #7<br><br>9'randomized controlled trial':ab,ti OR 'placebo':ab,ti OR 'double-blind':ab,ti10 'random':ab,ti OR 'placebo':ab,ti OR 'double-blind':ab,ti<br><br>10#8 AND #9
Web of Science	1 TS=(Spinal Muscular Atrophies of Childhood) OR TS=(Spinal Muscular Atrophy Type II) OR TS=(Spinal Muscular Atrophy, Type II) OR TS=(Spinal Muscular Atrophy, Infantile) OR TS=(Werdnig Hoffmann Disease) OR TS=(Spinal Muscular Atrophy Type I) OR TS=(SMA) OR TS=(muscular atrophy, spinal) OR TS=(Type I Spinal Muscular Atrophy) OR TS=(Spinal Muscular Atrophy) OR TS=(Juvenile Spinal Muscular Atrophy) OR TS=(Spinal Muscular Atrophy Type III) OR TS=(Muscular Atrophy, Juvenile) OR TS=(Kugelberg Welander Disease) OR TS=(Spinal Muscular Atrophy, Type 3) OR TS=(Muscular Atrophy, Spinal, Type III) OR TS=(Spinal Muscular Atrophy, Type IV) OR TS=(Spinal Muscular Atrophy, Adult Form)<br><br>2 TS=(nusinersen) OR TS=(ASO-10-27) OR TS=(ISIS-SMNRx) OR TS=(ISIS 396443) OR TS=(SPINRAZA) OR TS=(risdiplam)<br><br>3 #1 AND #2<br><br>4 TS=(random) OR TS=(placebo) OR TS=(double-blind) OR TS=(randomized controlled trial) OR TS=(randomized)<br><br>5 #3 AND #4
Cochrane	1 [Spinal Muscular Atrophies of Childhood]

	explode all trees<br><br>2 (Spinal Muscular Atrophies of Childhood):ti,ab,kw OR (Spinal Muscular Atrophy Type II):ti,ab,kw OR (Spinal Muscular Atrophy, Infantile):ti,ab,kw OR (Werdnig Hoffmann Disease):ti,ab,kw OR (Spinal Muscular Atrophy Type I):ti,ab,kw<br><br>3 (SMA):ti,ab,kw OR (muscular atrophy, spinal):ti,ab,kw OR (Type I Spinal Muscular Atrophy):ti,ab,kw OR (Spinal Muscular Atrophy):ti,ab,kw OR (Juvenile Spinal Muscular Atrophy):ti,ab,kw<br><br>4 (Spinal Muscular Atrophy Type III):ti,ab,kw OR (Muscular Atrophy, Juvenile):ti,ab,kw OR (Kugelberg Welander Disease):ti,ab,kw OR (Spinal Muscular Atrophy, Type 3):ti,ab,kw OR (Muscular Atrophy, Spinal, Type III):ti,ab,kw<br><br>5 (Spinal Muscular Atrophy, Type IV):ti,ab,kw OR (Spinal Muscular Atrophy, Adult Form):ti,ab,kw<br><br>6 (nusinersen):ti,ab,kw OR (risdiplam):ti,ab,kw<br><br>7 #1OR#2OR#3OR#4OR#5<br><br>8 #6AND#7

Figure S1. Funnel plots of (A) HFMSE, (B) MFM32, (C) RULM, (D)HINE-2, (E) SMAIS, (F) Adverse events, and (G) Severe adverse events.

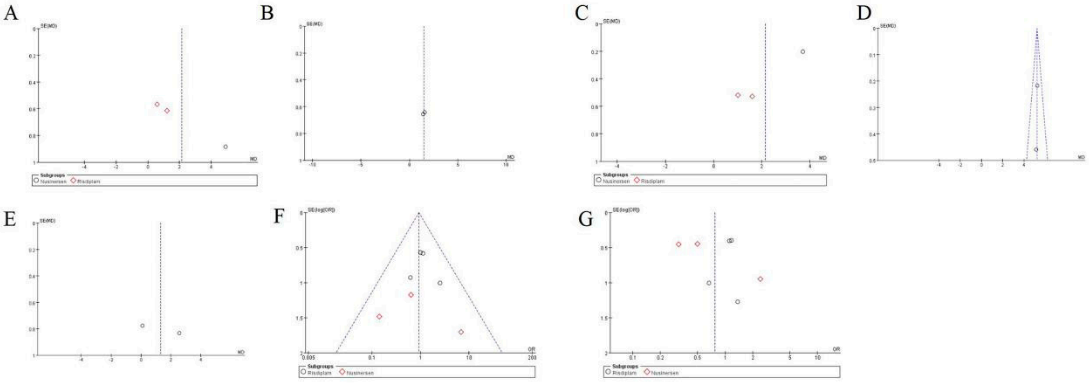

Figure S2. Sensitivity analysis of (A) HFMSE, (B) RULM, (C) SMAIS, and (D) Severe adverse events

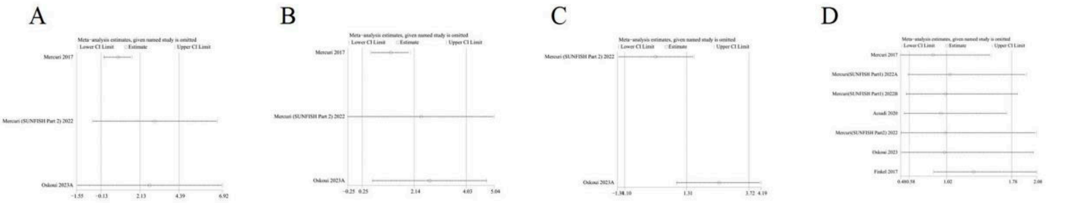

Supplement: Supplementary file 1 [file brainsci-13-01419-s001.zip › Supplementary Material.pdf]
